# From gut microbiota dysbiosis to immune dysregulation: pathogenic mechanisms and emerging therapeutic perspectives in pediatric asthma

**DOI:** 10.3389/fimmu.2026.1901737

**Published:** 2026-09-17

**Authors:** Zhimei Liu, Keying Yu, Jingjing Gao, Tanwen Zhang, Fenglu Liu, Shiwei Zhuang

**Affiliations:** 1 Department of Pediatrics, Affiliated Hospital of Changchun University of Chinese Medicine, Jilin, Changchun, China; 2 Cardiology Department, Affiliated Hospital of Changchun University of Chinese Medicine, Jilin, Changchun, China; 3 Department of Preventive Treatment, Affiliated Hospital of Changchun University of Chinese Medicine, Jilin, Changchun, China; 4 Traditional Chinese Medicine Department, JiLin Women and Children Health Hospital, Jilin, Changchun, China; 5 Department of Pediatrics, BaiCheng Traditional Chinese Medicine Hospital, Jilin, Baicheng, China; 6 Orthopedics Department, The Third Clinical Hospital Affiliated to Changchun University of Traditional Chinese Medicine, Jilin, Changchun, China

**Keywords:** airway inflammation, gut- lung axis, gut microbiota dysbiosis, immune dysregulation, pediatric asthma

## Abstract

Pediatric asthma is a chronic inflammatory airway disease with high heterogeneity. Its onset and progression are closely related to environmental exposures in early life and impaired immune regulation. Gut microbiota dysbiosis not only alters the production of metabolites such as short-chain fatty acids, 12,13-diHOME, and succinate, but also drives airway inflammation through multiple immunoregulatory mechanisms. Gut microbial dysbiosis can affect the production of metabolites, such as short-chain fatty acids, and promote airway inflammation through complex immune regulatory mechanisms. In addition, multi-axis interactions, including the gut-brain-lung and gut-bone-lung axis, may influence inflammatory phenotypes and disease heterogeneity in pediatric asthma. This review summarizes the characteristics of gut microbiota dysbiosis in pediatric asthma and focuses on the core mechanisms by which the gut–lung axis contributes to asthma pathogenesis, including metabolite-mediated regulation, disruption of immune homeostasis, mucosal barrier dysfunction, and multi-axis crosstalk. It also summarizes potential intervention approaches targeting the gut microbiota. Elucidating the gut microbiota–immune–multi-organ regulatory network may provide a theoretical basis for the early identification and precision intervention of pediatric asthma.

## 1 Introduction

Pediatric asthma is a chronic and heterogeneous respiratory disease characterized by airway inflammation, and it substantially affects children’s quality of life (1–3). Despite the reduction in the global age-standardized incidence rate of childhood asthma between 1990 and 2021, its disease burden is still high (4). In 2021, over 95.7 million children worldwide were living with asthma (5), and the global prevalence of childhood asthma was estimated at 10.2%. In children with asthma, eosinophilic inflammation is more common during both acute exacerbations and stable disease, whereas neutrophilic inflammation predominates in adults with acute asthma (6, 7). Therefore, the inflammatory mechanisms and related findings identified in adult asthma cannot be fully extrapolated to pediatric populations. Further research is needed to characterize the distinct immune and inflammatory features of pediatric asthma. Further research is needed to characterize the distinct immune and inflammatory features of pediatric asthma. Asthma pathogenesis involves a complex interplay among genetic susceptibility (8, 9), antibiotic exposure (10), and environmental factors (11). Immune dysregulation, including T-cell imbalance, also plays a critical role in the pathophysiology of asthma. Growing evidence suggests that gut microbiota dysbiosis and alterations in microbial metabolites may be important contributors to the development of pediatric asthma (12, 13).

The gut microbiota is a complex ecosystem composed of bacteria, fungi, and viruses. The composition of the pediatric gut microbiota is shaped by multiple factors, including delivery mode, feeding pattern, environmental exposures, and genetic background. Microbial communities such as *Firmicutes* and *Bacteroidetes* help maintain intestinal microbial homeostasis and host health in healthy gut. Their metabolites can also regulate distant organs through interconnected systems, including the gut–lung axis and the gut–brain axis (14). The gut–lung axis refers to the bidirectional relationship and functional interaction between the gut and the lungs. Gut microbial dysbiosis not only disrupts epithelial barrier integrity but also enables microbial metabolites to communicate bidirectionally with the respiratory tract through multiple regulatory networks, including circulatory and neural pathways (15). In addition, a reduced abundance of beneficial commensal bacteria, such as *Bifidobacterium* and *Bacteroides fragilis*, may disrupt immune homeostasis, promote neutrophil extracellular trap (NET) formation, and induce an imbalance in T helper (Th) cells polarization, thereby exacerbating airway inflammation. Therefore, further investigation of the gut–lung axis may not only help elucidate the pathogenesis of asthma but also provide new insights into the prevention and treatment of pediatric asthma.

Evidence from microbiome, immunological, and clinical studies remains fragmented and has not been systematically integrated. Consequently, how gut microbiota dysbiosis influences airway inflammation and pediatric asthma through immune and metabolic pathways remains incompletely understood. This review systematically summarizes alterations in the pulmonary and gut microbiota and their interactions in pediatric asthma. It further incorporates emerging evidence on microbial metabolites and multi-organ regulatory axes to expand the mechanistic framework of the gut–lung axis, with particular emphasis on the mechanisms through which gut–lung interactions contribute to asthma exacerbations. The review also evaluates the therapeutic potential and current limitations of gut microbiota-targeted interventions, thereby providing a more comprehensive understanding of the role of the microbiota in pediatric asthma.

## 2 Pediatric asthma

Asthma is a chronic inflammatory respiratory disease characterized by episodic bronchoconstriction and airway hyperresponsiveness, which lead to airflow limitation and symptoms such as wheezing, shortness of breath, chest tightness, and cough. These symptoms substantially impair the quality of life of affected children. As summarized in **Table 1**, investigating asthma endotypes and their associated pathogenic factors from the perspective of disease heterogeneity is important for elucidating disease mechanisms and developing early clinical interventions.

**Table 1:** Studies on pediatric asthma endotypes.

Asthma endotype or subgroup	Immunological and metabolic characteristics	Gut microbiota profiles	Ref.
T2-high endotype	Activation of Th2 cells and type 2 innate lymphoid cells; increased production of IL-4, IL-5, and IL-13; eosinophil maturation and recruitment; enhanced IgE production and STAT6 signaling; mucus hypersecretion and airway inflammation	Allergic asthma in children has been associated with reduced gut microbial diversity, increased relative abundance of Proteobacteria and Eggerthella, and decreased abundance of Megamonas, Lachnospiraceae CAG-56, and Prevotella. Increased Firmicutes and decreased Bacteroidetes have also been reported in children with allergic airway disease	(16–18)
Non-T2 endotype	Predominant Th1/Th17-associated immune responses; IL-6- and IL-17-mediated neutrophil recruitment; epithelial barrier disruption, mucus overproduction, and airway remodeling	Non-allergic asthma in children has been associated with reduced microbial diversity and broader alterations in gut microbial composition, including increased Proteobacteria, reduced Clostridia-related taxa, and enrichment of Coprobacillus, Niabella, Chitinophagales 37-13, and Microscillaceae OLB12	(16, 19, 20)
Metabotype 2	Lower neutrophil levels and serum inflammatory markers, accompanied by increased serum acylcarnitines and omega-3 fatty acids; associated with a relatively favorable clinical phenotype	Higher gut microbial richness and α-diversity; enrichment of Christensenellaceae R-7 group, Coprococcus, Oscillospiraceae NK4A214 group, Oscillospiraceae UCG-005, Anaerovoracaceae Family XIII AD3011 group, and Victivallis; reduced relative abundance of Lachnoclostridium	(16)
Metabotype 3	Reduced serum cholesteryl esters, phosphatidylcholines, and sphingomyelins; associated with a higher disease burden	Lower gut microbial richness and Shannon α-diversity, accompanied by a reduction in potentially beneficial bacteria and enrichment of Lachnoclostridium	(16)

### 2.1 Clinical endotypes of pediatric asthma

Asthma is a highly heterogeneous chronic inflammatory airway disease. The concept of “endotype,” proposed in 2008, has been used to explain this heterogeneity from the perspective of underlying pathobiological mechanisms (21). Based on immune characteristics, asthma can be broadly classified into type 2-high (T2-high) and non-type 2-high (non-T2) endotypes (22–24). In 2017, Israel et al. classified asthma into eosinophilic, neutrophilic, mixed granulocytic, and paucigranulocytic inflammatory phenotypes from the perspective of airway inflammation. This classification highlights the marked heterogeneity of asthma in terms of inflammatory characteristics (25–27), as shown in **Figure 1**.

**Figure 1:**
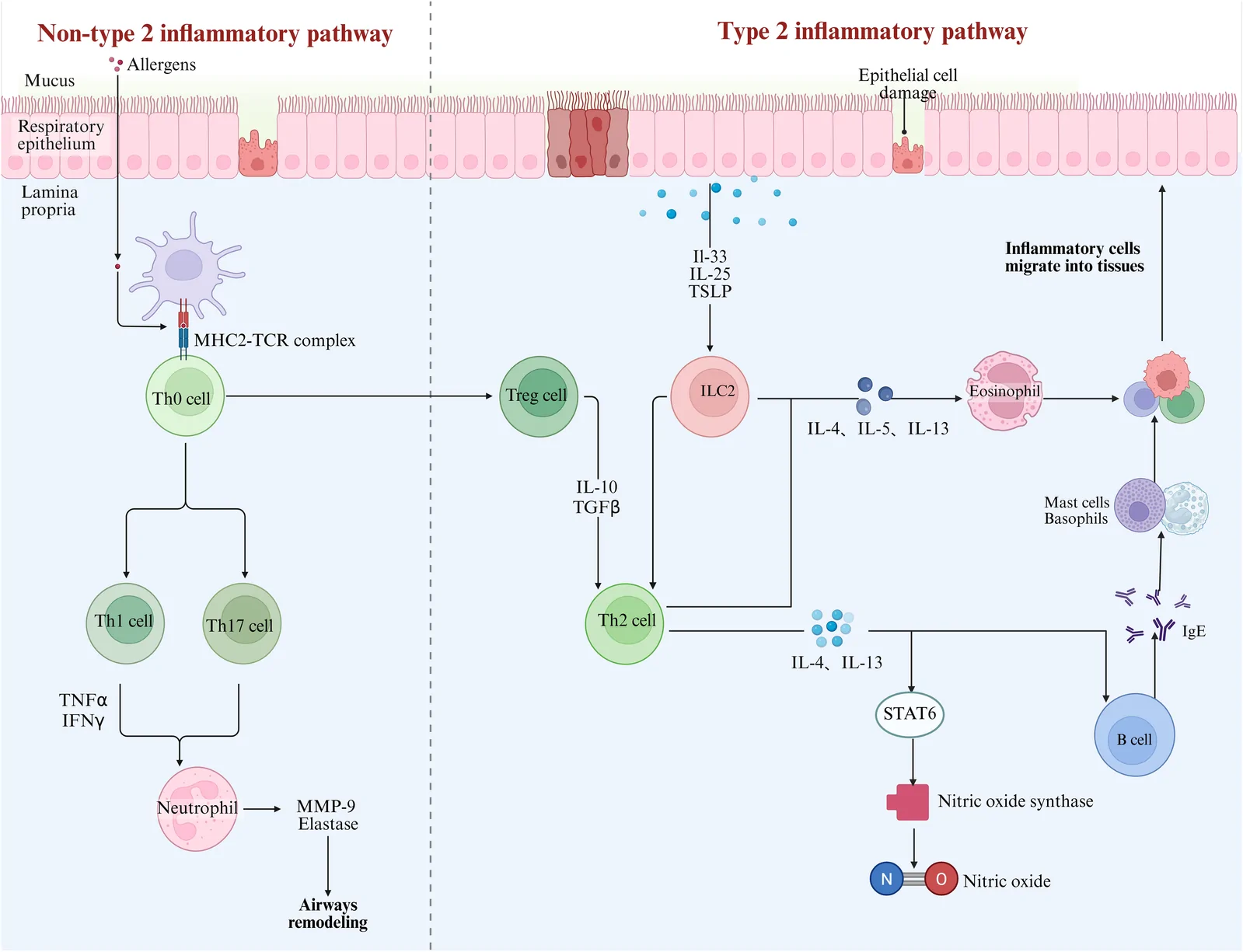
Phenotypes of pediatric asthma. T2-high asthma is mainly characterized by Th2/ILC2-mediated type 2 inflammation, with elevated levels of type 2 cytokines such as IL-4, IL-5, and IL-13, accompanied by eosinophilic inflammation and IgE-mediated allergic responses. Non-T2 asthma is primarily characterized by activation of Th1/Th17 immune pathways and neutrophilic inflammation.

Type 2-high asthma is one of the most common phenotypes of pediatric asthma. Its key feature is the activation of inflammatory pathways dominated by Th 2 cells and group 2 innate lymphoid cells (ILC2s) (18). These cells release type 2 cytokines, including IL-4, IL-5, and IL-13. These cytokines promote eosinophil maturation, recruitment, and activation, enhance IgE production by B cells, and activate signaling pathways such as STAT6 (28), thereby contributing to airway inflammation and mucus hypersecretion (29). In contrast to the T2-high endotype, which is typically dominated by eosinophilic inflammation, non-T2 asthma encompasses neutrophilic asthma characterized by Th1/Th17-mediated immune responses, as well as mixed granulocytic and paucigranulocytic asthma (19, 30). Driven by pro-inflammatory cytokines such as IL-6, CD4+ T cells differentiate into Th17 cells, which subsequently promote neutrophil recruitment. The resulting Th17 neutrophil inflammatory axis contributes to mucus hypersecretion, epithelial barrier disruption, and airway remodeling, thereby representing a key pathogenic mechanism underlying neutrophilic asthma (31, 32). Notably, different pediatric asthma endotypes may exhibit distinct gut microbiota profiles. A small cross-sectional study involving 57 children found reduced gut microbial diversity in children with asthma. Both allergic and non-allergic asthma were associated with an increased relative abundance of *Proteobacteria* and a decreased abundance of several genera within the class *Clostridia*. However, the specific microbial taxa involved differed between the two phenotypes, with more extensive alterations observed in children with non-allergic asthma. In children with allergic asthma, the relative abundance of *Eggerthella* was increased, whereas the abundances of *Megamonas*, Lachnospiraceae CAG-56, and *Prevotella* were decreased. In contrast, enrichment of taxa such as *Coprobacillus*, *Niabella*, Chitinophagales 37-13, and Microscillaceae OLB12 was observed exclusively in children with non-allergic asthma (33). Moreover, allergic airway diseases may be associated with distinct alterations in the gut microbiota, characterized by a higher relative abundance of *Firmicutes* and a significantly lower relative abundance of *Bacteroidetes* (17). Microbial alterations reported across studies are not entirely consistent, possibly because of differences in participant age, dietary patterns, geographic location, and sequencing methods. Therefore, no single bacterial phylum or genus can currently be regarded as a robust microbial biomarker for specific asthma endotypes. Greater attention should instead be paid to endotype-specific differences in the overall structure and functional characteristics of the gut microbial community.

Beyond allergic status, an integrated analysis of serum and fecal metabolomic profiles also identified distinct gut microbial patterns. Abdel-Aziz et al. classified 92 children with moderate-to-severe asthma into three metabotypes. Metabotype 2, which was associated with a more favorable clinical profile, exhibited greater gut microbial richness and α-diversity. It was enriched in *Christensenellaceae R27 group, Oscillospiraceae UCG-2005, Oscillospiraceae NK4A214 group, Coprococcus, Anaerovoracaceae Family XIII AD3011 group, and Victivallis*, but showed a lower relative abundance of *Lachnoclostridium*. In contrast, metabotype 3, which was characterized by a greater disease burden, showed reduced microbial richness and Shannon α diversity, together with a lower relative abundance of potentially beneficial bacteria and an enrichment of *Lachnoclostridium* (16).

Collectively, distinct pediatric asthma endotypes are associated with different gut microbiota profiles, and substantial microbial heterogeneity is also observed across metabotypes. However, studies directly comparing gut microbiota profiles across pediatric asthma endotypes remain limited, and the available findings may be influenced by differences in sample size, participant age, geographic location, and other potential confounding factors. Future studies should integrate metagenomic, metabolomic, and other multi-omics analyses into large-scale longitudinal pediatric cohorts to further clarify the potential role of the gut microbiota in the development and progression of distinct asthma endotypes.

### 2.2 Risk factors for pediatric asthma

Changes in the airway and gut microbiota in early life are significantly associated with asthma susceptibility and are regulated by host, environmental, and dietary factors (34). These factors may influence asthma risk by shaping early microbial colonization, immune system development, and the barrier function of the airway and intestinal mucosa, as shown in **Figure 2**.

**Figure 2:**
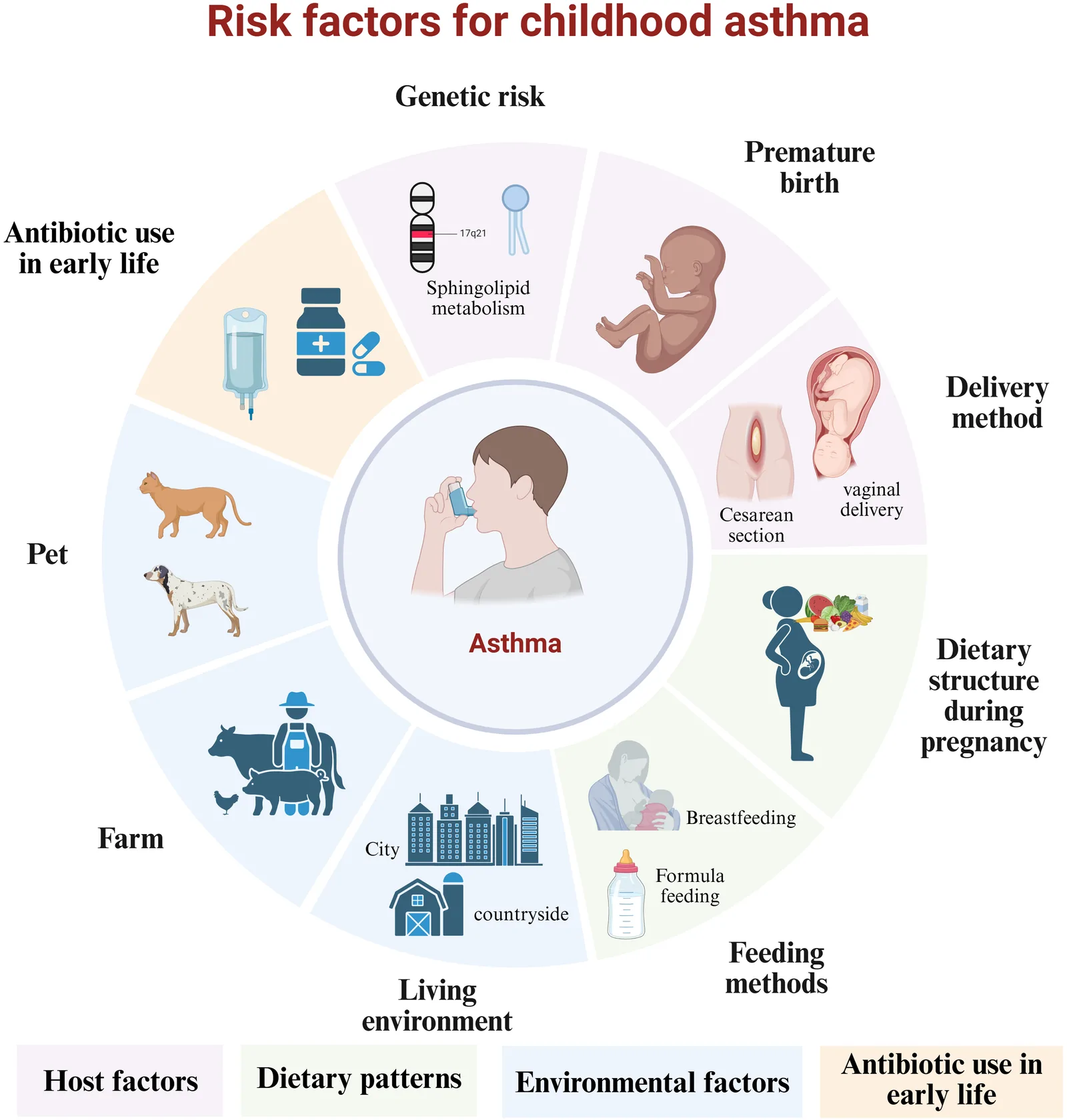
Risk factors for pediatric asthma. Host factors, dietary patterns, environmental exposures, and early-life antibiotic use are major risk factors for the development of pediatric asthma.

#### 2.2.1 Host factors

Numerous genomic studies have revealed genetic susceptibility to pediatric asthma. The 17q21 locus is one of the most prominent genetic risk factors for pediatric asthma and may contribute to disease susceptibility partly by regulating ORMDL3 expression (35, 36). Further studies suggest that this genetic susceptibility may contribute to early-onset asthma endotypes and alterations in airway function by affecting sphingolipid metabolism (37), endoplasmic reticulum stress, and the expression of inflammation-related genes (38). Gestational age and mode of delivery may modify the risk of childhood asthma by influencing gut microbial colonization during early life (39, 40). Preterm infants exhibit delayed gut microbiota maturation and delayed colonization by commensal bacteria, accompanied by increased relative abundances of potential pathogens such as *Klebsiella aerogenes* and *Staphylococcus epidermidis* (41). This microbial dysbiosis may disrupt immune system maturation and mucosal barrier development, while increasing susceptibility to lower respiratory tract infections, and may thereby be associated with an elevated risk of subsequent asthma. Mode of delivery also shapes the microbial exposures encountered by neonates during early life. Studies have shown that vaginally delivered infants have lower relative abundances of *Enterococcus* and *Klebsiella* spp. but a higher relative abundance of *Bifidobacterium* spp. in the gut microbiota. The sustained presence of these commensal bacteria is associated with greater microbiota maturity and a significantly lower long-term risk of asthma (42). Infants delivered by cesarean section exhibit delayed maturation of the respiratory microbiota and reduced colonization by commensal bacteria (43), together with an increased relative abundance of potentially pathogenic taxa, particularly within the phyla *Firmicutes* and *Proteobacteria* (42). This microbial dysbiosis is closely associated with an increased risk of childhood asthma.

#### 2.2.2 Dietary patterns

Maternal dietary patterns during pregnancy and postnatal feeding practices may influence susceptibility to childhood asthma by shaping the trajectory of microbial colonization during early life. A birth cohort study using 16S rRNA gene sequencing found that maternal fruit intake was associated with the structure of the infant gut microbial community (44). Animal studies further showed that a maternal low-fiber diet reduced the secretion of Flt3L by the intestinal epithelium of the offspring. This reduction delayed plasmacytoid dendritic cell development and the establishment of immune tolerance, thereby increasing susceptibility to lower respiratory tract infections (45).

Human milk contains viable bacteria of both endogenous and exogenous origin, which may play an important role in shaping the gut microbiota of infants. The relative abundances of *Streptococcus* spp. and *Veillonella dispar* in human milk were positively correlated with their abundances in the infant gut, suggesting that human milk may serve as a source of bacteria that contributes to early gut microbial colonization (46). The abundances of *Bifidobacterium*, *Staphylococcus*, and *Streptococcus* decrease after the cessation of breastfeeding, whereas members of the family Lachnospiraceae gradually increase (47). These changes further reflect the important role of breastfeeding in establishing the infant gut microbiota. Commensal bacteria can produce short-chain fatty acids (SCFAs), which reduce excessive Th2 inflammation by promoting the development of regulatory T cells (Tregs). This process is considered one of the key mechanisms underlying the protective effect of breastfeeding against asthma (13, 46). In addition, breastfeeding may mitigate the functional disruption of the gut microbiota caused by antibiotic exposure during infancy. This protective effect may be associated with the enrichment of *Bifidobacterium longum* subsp. *infantis* and a lower risk of antibiotic-associated childhood asthma. Moreover, fucosylated human milk oligosaccharides in breast milk are positively associated with the abundance of this bacterium and related changes in microbial functions (48).

#### 2.2.3 Environmental factors

Early-life environmental exposures may influence susceptibility to childhood asthma by altering the colonization and maturation trajectories of the infant gut microbiota. Rural and farm environments harbor greater microbial diversity than urban environments (49). Children raised in these environments have a significantly lower incidence of asthma and allergic diseases (50, 51). The PASTURE birth cohort study found that farm exposure was associated with accelerated gut microbiota maturation by 12 months of age. Greater microbiota maturity was, in turn, associated with a lower risk of asthma at school age (52, 53). However, the effects of environmental factors on pediatric asthma are not unidirectional. Environmental stressors, including urbanization and air pollution, may increase asthma risk by disrupting the stability of the gut and respiratory microbiota. The MAMI birth cohort study showed that chronic exposure to NO_2_ was associated with reduced α-diversity of the infant gut microbiota and increased relative abundances of *Haemophilus*, *Akkermansia*, *Alistipes*, *Eggerthella*, and *Tyzzerella* (54). Household pet exposure has also been associated with alterations in the composition and developmental trajectory of the infant gut microbiota (55, 56), including greater richness of *Bifidobacterium* and Firmicutes taxa and enrichment of *Ruminococcus* and *Oscillospira* (57–59). These findings further support a close association between environmental exposures and the development and progression of childhood asthma.

#### 2.2.4 Early-life antibiotic use

Early-life antibiotic exposure may increase susceptibility to childhood asthma by disrupting the normal establishment of the infant gut microbiota. The Canadian CHILD birth cohort study suggested that the recent decline in childhood asthma incidence may be associated with more judicious antibiotic use during infancy (60). Further microbiome analyses showed that, among infants who were not breastfed, antibiotic exposure was associated with a threefold higher risk of childhood asthma, together with widespread disruptions in the taxonomic composition and metabolic functions of the gut microbiota (48). Multiple infant cohort studies and clinical trials have further shown that the effects of antibiotic exposure on the early-life gut microbiota may persist over time. Treatment with amoxicillin or macrolides during infancy was associated with altered gut abundances of *Bifidobacterium*, *Enterobacter*, and *Clostridium* (61). Broad-spectrum antibiotic treatment during the neonatal period may reduce the gut abundance of *Bifidobacterium* while increasing the abundances of *Klebsiella* and *Enterococcus* compared with those in untreated controls (62). Animal studies have provided further mechanistic evidence for the effects of antibiotics on the gut microbiota. Early-life exposure to azithromycin or amoxicillin disrupted the abundance of gastrointestinal microbial taxa and microbial metabolite production. Following exposure to environmental allergens such as house dust mite, these alterations were associated with elevated IgE and IL-13 levels, excessive activation of Th2/Th17 immune pathways, and increased airway hyperresponsiveness (63). Therefore, early-life antibiotic exposure may increase the risk of allergic airway inflammation and childhood asthma by persistently disrupting the gut microbiota and its immunoregulatory functions.

## 3 Gut microbiota dysbiosis in pediatric asthma

The gut and respiratory tract harbor distinct yet interconnected microbial communities that collectively form the microbial basis of the gut–lung axis. Alterations in the microbiota at either site may be associated with asthma-related inflammation in children. The airway microbiota reflects the local microbial environment at the primary site of asthma pathology, whereas the gut microbiota may influence respiratory health through the gut–lung axis.

### 3.1 Airway microbiota in asthma

A healthy airway microbiota plays an important role in maintaining airway homeostasis and regulating immune responses, and its composition exhibits marked spatial heterogeneity. Distinct microenvironments across different regions of the respiratory tract shape site-specific microbial communities. The nasopharynx is predominantly colonized by *Moraxella*, *Staphylococcus*, *Corynebacterium*, *Haemophilus*, and *Streptococcus*, whereas the oropharynx is enriched in *Prevotella*, *Veillonella*, and *Streptococcus* (64).

Compared with healthy children, children with asthma exhibit a certain degree of ecological imbalance in the lung microbiota (65). In the upper respiratory tract, the nasal microbiota of children with asthma is enriched in *Moraxella*. Its relative abundance is positively associated with an increased frequency of asthma exacerbations, a finding that has been replicated in multiple population-based cohorts (66, 67). In contrast, enrichment of certain commensal bacteria, such as *Corynebacterium* and *Dolosigranulum*, is associated with a lower proportion of eosinophils and fewer asthma exacerbations. These findings suggest that distinct bacterial communities may influence asthma phenotypes by modulating the local immune microenvironment (68, 69). Studies on early asymptomatic airway colonization have further shown that colonization by *Streptococcus pneumoniae* and *Haemophilus influenzae* during early life is associated with a significantly increased risk of asthma by the age of 5 years. This effect appears to be more pronounced in children with atopy (68, 70). Viruses and fungi play important roles in asthma pathogenesis, with respiratory syncytial virus (RSV) and rhinovirus (RV) being particularly well studied. Prospective birth cohort studies have shown that children who experience RSV infection during infancy have a significantly higher incidence of asthma by the age of 5 years (71). In addition, lower respiratory tract RV infection in early life may increase the risk of asthma before the age of 6 years by several dozen-fold (72). RV infection may also increase the susceptibility of children with atopy to airway inflammation by promoting the expression of airway remodeling-related factors, such as lumican and collagen, and by prolonging bronchial hyperresponsiveness, thereby aggravating disease progression (73, 74). Although the role of fungal communities in asthma remains inconclusive, several studies have reported alterations in the airway mycobiome of patients with asthma (75).

The healthy airway microbiota exhibits distinct spatial heterogeneity, and alterations in the airway microbiota are closely associated with childhood asthma risk, exacerbation frequency, and disease progression. However, the airway microbiota should not be regarded as an isolated determinant of the disease. Rather, it represents the pulmonary component of the broader gut–lung axis and may be influenced by systemic immune and metabolic signals originating from the gut.

### 3.2 Gut microbiota in asthma

The establishment of a healthy gut microbiota is essential for the development of immune homeostasis, especially during early life. Early microbial colonization contributes to immune system maturation and respiratory health in infants and young children. The neonatal gut undergoes rapid microbial colonization after birth and is initially dominated by facultative anaerobes, such as Enterococcus, Escherichia, and Streptococcus (76). As feeding patterns, environmental exposures, and host development progress, the gut is gradually colonized by anaerobic bacteria, including *Bifidobacterium*, *Lactobacillus*, and members of the family Clostridiaceae within the phylum Firmicutes (77, 78). These dominant bacterial taxa not only enhance gut microbial diversity and stability but also provide essential stimuli for immune system maturation. Longitudinal studies have shown that gut microbial diversity gradually increases in healthy infants, and the stable colonization of SCFAs-producing bacteria, such as *Faecalibacterium* and *Roseburia*, is an important marker of intestinal ecological maturation (79). Although the neonatal gut virome has a low biomass, it may regulate the population structure of commensal microbial communities through niche competition (80), thereby contributing to host health.

Gut microbiota dysbiosis may play an important role in the onset, recurrence, and severity of pediatric asthma, and characteristic alterations in bacterial communities have been confirmed by multiple cohort studies. A cohort study showed that reduced abundances of *Faecalibacterium*, *Lachnospira*, *Rothia*, and *Veillonella* in fecal samples from 3-month-old infants were associated with atopy and wheezing at 1 year of age (79). Similarly, Canadian and Australian cohort studies have shown that high-risk infants exhibit a reduced abundance of SCFAs producing taxa in the gut, accompanied by an increase in opportunistic bacteria, including members of the family Enterobacteriaceae and some Clostridiaceae taxa. These alterations are closely associated with asthma and recurrent wheezing during follow-up (81). These data suggest that microbiota dysbiosis in pediatric asthma involves not only a reduction in overall microbial diversity but also the depletion or imbalance of key microbial taxa. In addition, the gut mycobiome and virome may also play important roles in pediatric asthma. Fujimura et al. reported that infants at higher risk of asthma had increased relative abundances of *Candida* and *Rhodotorula* in the gut, along with a reduced abundance of *Malassezia* (79). Reduced bacteriophage diversity during infancy is associated with an increased risk of asthma, raising the possibility that the gut virome participates in asthma development through its effects on bacterial communities and host immune responses.

Emerging evidence from animal studies suggests that alterations in the gut microbiota may be accompanied by changes in the respiratory microbiota. In an ovalbumin-induced mouse model of asthma, fecal microbiota transplantation altered the β-diversity of both the gut and respiratory microbiota, reduced the relative abundance of Proteobacteria, and partially reversed asthma-associated microbial dysbiosis. These microbial changes were accompanied by attenuated airway inflammation and reduced levels of IgE and proinflammatory cytokines (82). Conversely, pulmonary inflammation may also disrupt gut microbiota homeostasis. Allergic airway inflammation induced by ovalbumin or house dust mite was accompanied by alterations in the gut microbiota (83, 84). Respiratory viral infections may also alter the composition of the gut microbiota and the intestinal microenvironment (85, 86). These studies support a potential bidirectional relationship between the gut and respiratory microbial ecosystems. However, direct evidence that the gut and airway microbiota mutually reshape each other remains limited, particularly in children. Further investigation into the mechanisms underlying the gut–lung axis may therefore help clarify how interactions between the gut and respiratory microbiota contribute to the development and progression of childhood asthma.

## 4 Gut–lung axis–mediated mechanisms in asthma pathogenesis

The contribution of the gut–lung axis to asthma pathogenesis is not mediated by a single process. Microbial metabolites, immune homeostasis, mucosal barrier dysfunction, and inter-axis crosstalk form an interconnected regulatory network. Gut microbiota dysbiosis may modulate host immune responses and compromise mucosal barrier integrity by altering microbial metabolites, including short-chain fatty acids, tryptophan derived metabolites, and bile acids. Barrier disruption may, in turn, facilitate the entry of microbe-derived molecules and metabolites into the systemic circulation, further disturbing immune homeostasis and the pulmonary immune microenvironment. Moreover, the gut–lung axis may interact with other organ axes to jointly regulate the transmission of inflammatory and immune signals.

### 4.1 Microbial metabolites

Metabolites produced by the respiratory microbiota are important in maintaining airway immune homeostasis and barrier integrity. Through the gut–lung axis, various metabolites synthesized by the gut microbiota can act as key signaling mediators in the regulation of pulmonary immunity (87). Related metabolites, including SCFAs, bile acids (BAs), and tryptophan-derived metabolites, can cross the intestinal epithelial barrier, enter the systemic circulation, and regulate immune cell function and inflammatory responses in distal lung tissues (88). These representative microbiota-derived metabolites and their immunoregulatory effects are summarized in **Table 2**.

**Table 2:** Microbial metabolites associated with the gut–lung axis and their immunoregulatory effects.

Microbial metabolite	Mechanisms/signaling pathways	Immunoregulatory effects	Evidence in pediatric asthma	Refs.
SCFAs	Inhibition of HDACs; activation of GPR41/GPR43; modulation of the p38 MAPK/NF-κB pathway	Promote Treg differentiation and recruitment; suppress Th2-mediated inflammation and eosinophil infiltration; reduce IgE production; attenuate airway inflammation and hyperresponsiveness	Higher butyrate and propionate levels were associated with a reduced risk of pediatric asthma	(89–91)
Indoles, tryptamine, Kyn	Activation of AhR; involvement of the IDO–Kyn pathway; inhibition of NF-κB/NLRP3 signaling	Enhance mucosal barrier function; induce tolerogenic DCs and Tregs; suppress type 2 inflammation; maintain airway immune tolerance	Exert protective effects against pediatric asthma by promoting immune tolerance and suppressing airway inflammation	(92–95)
5-HT	Platelet release	Promote allergic airway inflammation and aggravate asthma pathology	Promote pediatric asthma by enhancing allergic airway inflammation	(96)
BAs	Activation of FXR signaling; regulation of the UPR–ATF6α pathway; modulation of DC function	Suppress eosinophilic inflammation; alleviate endoplasmic reticulum stress; reduce Th2-related cytokines and IgE levels; ameliorate airway inflammation and remodeling	Altered secondary bile acid metabolism is associated with pediatric asthma, while beneficial bile acids may alleviate airway inflammation	(97–100)
Other gut microbiota-derived metabolites	PPARγ-related signaling, protein succinylation, and polyamine/histamine-mediated pathways	Modulate immune tolerance and airway inflammation	Elevated neonatal 12,13-diHOME is associated with increased asthma risk; succinate is elevated in pediatric asthma	(101–103)

#### 4.1.1 SCFAs

The gut microbiome communicates with the respiratory system through metabolite-mediated signaling pathways. Among these metabolites, Short-chain fatty acids (SCFAs) are considered one of the key functional mediators of the gut–lung axis (104). Commensal gut bacteria generate SCFAs through dietary fiber fermentation. These metabolites support intestinal epithelial cell metabolism and may reach distal organs through the systemic circulation, where they contribute to immune homeostasis in tissues such as the lungs and brain.

Population-based studies have linked insufficient SCFA availability in early life to a higher risk of asthma and atopic disease. Lower SCFA levels in infancy were associated with associated with an increased risk of asthma later in life (104–107). while children with persistent asthma generally exhibit reduced fecal SCFA concentrations during the disease course (108). Evidence from animal models further supports a causal role for SCFA deficiency in asthma development. Correction of gut SCFA imbalance by fecal microbiota transplantation (FMT) in asthmatic rat models markedly attenuates airway inflammation and improves asthma-like symptoms (109). Prenatal antibiotic exposure reduces butyrate levels, suppresses type I interferon signaling in ILC2s, and induces epigenetic remodeling. This metabolic and immune disturbance promotes abnormal expansion and functional activation of pulmonary ILC2s in neonates, thereby exacerbating allergic airway inflammation in adulthood (110). This observation links microbiota-driven immune pathways to the development of asthma. Among SCFAs, butyrate is considered the most potent inhibitor of allergic immune responses in the lung (111). *In vivo* studies have shown that butyrate effectively suppresses eosinophil infiltration in the lungs of asthmatic mice (89). Propionate, in contrast, promotes the recruitment of intestinal Tregs to the lung, thereby limiting excessive Th2-mediated inflammation (91). The European PASTURE cohort further showed that higher fecal levels of butyrate and propionate during infancy were associated with a reduced risk of asthma (89).

At the molecular level, SCFAs exert immunoregulatory effects through multiple signaling pathways. SCFAs can inhibit histone deacetylases or activate G protein-coupled receptors, such as GPR41 and GPR43, thereby promoting Treg differentiation and maintaining immune tolerance. On the other hand, they can suppress eosinophil recruitment and attenuate Th2-driven allergic inflammation (90, 112). Notably, butyrate also suppresses the p38 MAPK/NF-κB signaling pathway in follicular helper T cells in a GPR43-dependent manner, thereby reducing IgE production and asthma exacerbation (113). In addition, SCFAs can directly regulate the epigenetic program of B cells by inhibiting histone deacetylases, thereby modulating antibody production and autoimmune responses (114). Moreover, reduced fecal butyrate levels have been associated with increased house dust mite-specific IgE levels in mice and a higher risk of asthma in early childhood (115), further supporting the key role of butyrate in the immune regulation of asthma. Taken together, SCFAs are key microbial metabolites through which the gut microbiota exert protective effects against asthma via the gut–lung axis. Enhancing SCFA production in early life through dietary modulation or microbiota-targeted strategies may provide a new approach for the early prevention and intervention of asthma.

#### 4.1.2 Tryptophan

Tryptophan (Trp) is an essential aromatic amino acid that is mainly obtained from the diet and plays a central role in regulating host metabolism and immune homeostasis (116). Beyond host-intrinsic metabolism, the gut microbiota can metabolize tryptophan to generate a variety of immunologically active metabolites (117). For example, *Escherichia coli* and *Lactobacillus gasseri* can convert tryptophan into indole, whereas *Clostridium sporogenes* produces tryptamine. These microbiota-derived tryptophan metabolites serve as important signaling molecules through which the gut microbiota influence immune function in distal organs. In recent years, tryptophan metabolism has been recognized as one of the key pathways through which the gut microbiota regulate airway immune tolerance via the gut–lung axis (118). Trp metabolism mainly occurs through the serotonin, indole, and kynurenine (Kyn) pathways. The Kyn pathway is the major route of tryptophan catabolism. Metabolites from these pathways participate in gut–lung axis-mediated immune regulation through key molecules such as the aryl hydrocarbon receptor (AhR) and indoleamine 2,3-dioxygenase (IDO).

The AhR is a ligand-dependent transcription factor involved in the immune regulation of allergic asthma. Microbiota-derived indole tryptophan metabolites can act as endogenous ligands of AhR. These metabolites promote IL-22 secretion by innate lymphoid cells through AhR activation and inhibit the NF-κB/NLRP3 signaling pathway, thereby enhancing mucosal barrier function and maintaining immune tolerance (92). AhR activation can induce tolerogenic dendritic cells (DCs) and promote the differentiation of Foxp3^+^ Tregs, which may attenuate airway hyperresponsiveness (119). Such AhR activation mediated by microbiota-derived tryptophan metabolites exerts anti-inflammatory and protective effects. In contrast, environmental pollutants such as benzo[a]pyrene can aberrantly activate AhR and drive interactions between airway epithelial cells and fibroblasts. This mechanism promotes fibroblast activation and collagen deposition, thereby aggravating airway remodeling and asthma progression (94).

The IDO mediated Kyn pathway represents another key mechanism by which tryptophan metabolism regulates immune tolerance. IDO catalyzes the conversion of tryptophan into Kyn. This process suppresses T-cell proliferation by reducing tryptophan availability and (94), at the same time, promotes the differentiation of Tregs while inhibiting Th2-type inflammatory responses (120). Clinical studies have shown that IDO levels in the peripheral blood and sputum of patients with allergic asthma are significantly lower than those in healthy controls. This finding provides important evidence that IDO is involved in the inflammatory regulation of pediatric asthma (121). In allergic asthma, an imbalance between type 2 and type 1 cytokines can suppress IDO1 expression in airway epithelial cells and impair the Kyn pathway of tryptophan metabolism, thereby further aggravating asthma (122). Therefore, IDO may represent an important therapeutic target for asthma regulation (122). Importantly, IDO expression is regulated by interferon-γ (IFN-γ). As a key Th1 cytokine, IFN-γ not only contributes to antiviral and antibacterial defense but also induces IDO expression in antigen-presenting cells (95). This process helps establish an immune-tolerant microenvironment that suppresses allergic airway inflammation (95). IFN-γ is a critical cytokine in antiviral and antibacterial defense. It can further suppress asthmatic inflammation by regulating the Th1/Th2 balance (123). The serotonin pathway represents another mechanism by which tryptophan metabolism is linked to asthma pathogenesis. Serotonin (5-hydroxytryptamine, 5-HT) is a classical neurotransmitter in the central nervous system and also functions as a peripheral immunomodulatory mediator. Its production is regulated by tryptophan hydroxylase 1, and platelet-derived serotonin can promote allergic inflammation in the airways (96).

Tryptophan metabolites are important mediators of the gut–lung axis by linking the gut microbial ecosystem with the host immune system. Strategies that modulate microbial tryptophan metabolism may provide a useful approach for regulating airway inflammation and improving asthma prevention and treatment.

#### 4.1.3 Bile acids

BAs are important products of cholesterol metabolism and key immunological signaling molecules in the gut–lung axis. Primary bile acids are synthesized from cholesterol in host hepatocytes and mainly include cholic acid and chenodeoxycholic acid. These primary BAs are subsequently transformed by the gut microbiota into secondary BAs, such as ursodeoxycholic acid (UDCA) (97, 124).

Secondary BAs exhibit pronounced biological activity and can directly shape the gut microbial ecosystem. Their antimicrobial effects may selectively inhibit certain microbial taxa while favoring bile acid-tolerant bacteria, thereby modulating the composition of the gut microbiota (125, 126). Therefore, alterations in the bile acid metabolic profile not only reflect the functional status of the microbiota but also, in turn, shape the composition of the microbial ecosystem. UDCA is a secondary BAs generated through gut microbiota-mediated metabolism and is recognized as a beneficial bile acid with immunomodulatory properties. Studies have shown that UDCA has anti-inflammatory and immune homeostasis-regulating properties. It can modulate mucosal immune responses by reducing extraintestinal eosinophil-mediated inflammation, promoting IL-12 production, and increasing the migration and antigen-presenting capacity of DCs (98). Therefore, bile acid signaling may regulate pulmonary immune function through the gut–lung axis. Compared with children with asthma alone, children with asthma and comorbid food allergy exhibit BA metabolic remodeling, characterized by enhanced microbiota-mediated production of secondary BAs and altered bile acid conjugation patterns (127). This altered metabolic profile reflects a functional shift in the gut microbiota and supports the potential use of BAs as biomarkers for food allergy-associated asthma (128).

Further mechanistic studies have shown that several conjugated BAs can directly regulate cellular stress pathways associated with airway inflammation. Conjugated BAs, such as alanyl-β-muricholic acid, can preferentially activate the ATF6α branch of the unfolded protein response (UPR). This activation suppresses allergen-induced endoplasmic reticulum stress, airway inflammation, and airway hyperresponsiveness, thereby alleviating allergic asthma (99). Meanwhile, the role of specific beneficial microbial taxa in bile acid-mediated immune regulation has also been clarified. Beneficial taxa such as *Candidatus Arthromitus* and *Ligilactobacillus* can upregulate farnesoid X receptor (FXR) protein expression, remodel the bile acid metabolic profile, and activate Treg-associated immune pathways. These effects effectively suppress airway inflammation and tissue injury, thereby improving asthma-related phenotypes (100). These findings further support an important role for the gut microbiota–bile acid–immune regulation axis in the development and progression of asthma. In addition, bile acid derivatives play an important role in regulating Th2-type inflammation. Tauroursodeoxycholic acid can significantly reduce house dust mite induced levels of IL-4, IL-5, IL-13, and IgE, whereas β-muricholic acid suppresses the production of IL-6 and IL-33 (99).

Taken together, BAs play an important role in the immunoregulation of pediatric asthma. A deeper understanding of their functions in metabolic regulation, immune signaling, and interorgan crosstalk may help bridge the gap between taxonomic alterations in the gut microbiota and disease mechanisms. This knowledge may also provide a theoretical basis and potential therapeutic targets for precision interventions in pediatric asthma.

#### 4.1.4 Other gut microbiota-derived metabolites

In addition to SCFAs, BAs, and tryptophan metabolites, the gut microbiota may contribute to gut–lung axis mediated childhood asthma by modulating other metabolites. Among these, the linoleic acid-derived oxylipin 12,13-dihydroxy-9Z-octadecenoic acid (12,13-diHOME) may represent an important microbial metabolite linking neonatal gut microbiota dysbiosis to the risk of childhood asthma (101). In two independent cohort studies, elevated levels of bacterially derived 12,13-diHOME in neonatal stool were associated with an increased likelihood of developing atopic disease and asthma during childhood. Further studies have shown that epoxide hydrolases derived from gut bacteria can promote the production of 12,13-diHOME. Elevated 12,13-diHOME levels may alter PPARγ-related signaling, suppress the secretion of anti-inflammatory cytokines, and reduce regulatory T-cell abundance, thereby impairing immune tolerance and promoting allergic inflammation in the lungs (102).

Succinate is a key intermediate in energy metabolism and an important immunoregulatory signaling molecule. It can be produced through the host tricarboxylic acid cycle or generated by the metabolism of certain gut microorganisms (129, 130). Urinary metabolomic studies of childhood asthma have identified succinate as one of the metabolites most strongly enriched in the urine of children with asthma (131). However, these studies cannot determine whether the detected succinate originates from host metabolism or the gut microbiota. Interestingly, multilevel evidence from patients with allergic airway inflammation (AAI), animal models, and cell-based experiments suggests that gut microbiota-derived succinate may aggravate allergic airway inflammation by promoting protein succinylation in lung tissue. In contrast, fecal microbiota transplantation reduced succinate levels and protein succinylation, accompanied by attenuated airway inflammation (103). The underlying mechanism may involve succinate-induced protein succinylation in lung tissue, particularly the succinylation of SOD2 K68. Therefore, succinate may serve as an important metabolic signal linking gut microbiota dysmetabolism to pulmonary immune inflammation. However, current studies of childhood asthma have mainly documented altered succinate metabolism. Whether gut microbiota-derived succinate directly contributes to childhood asthma remains to be established through longitudinal pediatric cohort studies and targeted metabolomic analyses. Metabolites such as spermidine, spermine, and microbially derived histamine may also participate in gut–lung axis-related immune regulation. Existing evidence suggests that these metabolites may be associated with airway inflammation, immune dysregulation, and distinct childhood asthma phenotypes (33, 132, 133).

### 4.2 Disruption of immune homeostasis

Asthma is a heterogeneous disease characterized by chronic airway inflammation, and its development and progression are closely associated with dysregulated immune regulation. Alterations in the gut microbiota and its metabolites may further disrupt host immune homeostasis and thereby influence airway inflammatory phenotypes through the gut–lung axis. Among the immune mechanisms involved, T-cell dysregulation and neutrophil-mediated inflammatory responses appear to be particularly important.

#### 4.2.1 T cells

The fetal and perinatal periods are essential for immune system development and the establishment of immune tolerance. The intrauterine environment has been considered relatively sterile, the host is rapidly exposed to external microbial antigens after birth (134). Early-life microbial colonization contributes to the regulation of CD4^+^ T-cell differentiation. Microbial signals under physiological conditions can induce the production of Th1-type cytokines, such as IL-2, and promote the differentiation of naïve Th0 cells into Th1 cells (96). This immune shift helps maintain the Th1/Th2 immune balance and limits excessive Th2 activation. If this immune transition is delayed or impaired in early life, Th2 responses may dominate and increase the risk of atopic diseases. Therefore, Th1/Th2 imbalance is regarded as one of the key immunological mechanisms underlying the development and progression of asthma. Beyond the classical Th1/Th2 immune axis, Fujimura et al. reported that Th17/Treg imbalance is closely associated with moderate-to-severe asthma (79). This association is particularly evident in asthma phenotypes characterized by neutrophilic inflammation (135).

Pediatric studies have provided preliminary evidence of an association between the early-life gut microbiota and altered T-cell immune responses (136). Fujimura et al. found that a distinct gut microbiota state during the neonatal period was associated with an increased risk of subsequent multisensitized atopy and childhood asthma. *In vitro*, fecal water from high-risk infants increased the proportion of IL-4^+^CD4^+^ T cells and reduced the proportion of CD4^+^CD25^+^Foxp3^+^ regulatory T cells, suggesting that the early-life gut microbiota may influence T-cell differentiation (79). Henrick et al. further found that supplementation with *Bifidobacterium longum* subsp. *infantis* in infants increased gut-derived indole-3-lactic acid levels and was accompanied by reduced Th2- and Th17-associated inflammatory signaling (13). However, asthma was not included as a clinical endpoint in this study. Therefore, the findings primarily support a role for early microbial exposure in shaping immune development, rather than directly demonstrating a preventive effect against childhood asthma. Hu et al. observed an elevated Th17/Treg ratio, increased levels of IL-17 and IL-6, and decreased levels of IL-10 and TGF-β in children with allergic asthma. They also found that reduced IDO activity was associated with Th17/Treg imbalance (137). These findings support the presence of T-cell abnormalities in childhood asthma. However, because the gut microbiota was not assessed, it remains unclear whether these immune alterations were directly driven by gut microbiota dysbiosis.

Animal and *in vitro* studies have further revealed potential regulatory mechanisms. Specific strains of *Bifidobacterium* and *Lactobacillus* may modulate dendritic cell function, enhance IL-12 and IFN-γ production, and suppress Th2- and IgE-associated responses (138). Supplementation with *Lactobacillus johnsonii* or *Lactobacillus reuteri* attenuated allergen-induced Th2 inflammation, reduced IgE levels, and improved airway hyperresponsiveness (139). In addition to microbial cellular components, microbial metabolites are also important mediators of T-cell regulation. SCFAs can promote the generation of peripheral regulatory T cells and enhance their suppressive function (140–144). Gut bacteria-derived serotonin may promote early Treg expansion (145), whereas polysaccharide A from *Bacteroides fragilis* can increase IL-10 and Foxp3 expression (146). In addition, *Bifidobacterium* derived indole-3-lactic acid may suppress Th2 and Th17 responses. Therefore, regulatory T cells may serve as important cellular mediators through which gut microorganisms modulate immune responses in childhood asthma via the gut–lung axis. Animal studies have shown that gut microbiota induced Th17 cells can acquire tissue-migratory capacity and participate in pulmonary immune responses, suggesting that T cells may act as an important cellular link between the gut and the lungs (147, 148).

T cells are central to immune regulation of pediatric asthma. The stability of the Th1/Th2 and Th17/Treg immune axes is essential for maintaining immune tolerance and restraining airway inflammation. However, current findings appear to depend substantially on the microbial strain, dose, host condition, and immune environment. Future longitudinal, multicenter pediatric cohort studies should integrate metagenomic and metabolomic data, T-cell phenotypes, and clinical outcomes to identify the microbial–immune mechanisms that drive specific asthma endotypes.

#### 4.2.2 Neutrophil

Neutrophils originate from the bone marrow and serve as rapidly responding effector cells in the innate immune response. Neutrophils can be extensively recruited to the intestinal mucosa during intestinal inflammation, and their excessive accumulation and release of pro-inflammatory mediators can aggravate mucosal barrier damage and amplify local inflammatory responses (149). Early clinical studies using induced sputum found that children with non-atopic asthma more commonly exhibited neutrophilic airway inflammation (150). Another case-control study found elevated sputum levels of neutrophil activation markers, including myeloperoxidase and human neutrophil lipocalin, in children with asthma, particularly those with moderate-to-severe persistent asthma. Higher levels of these markers were also associated with impaired lung function and greater disease severity (151). These findings suggest a potential pathogenic role for neutrophils in pediatric asthma. However, clinical evidence regarding the interactions between neutrophils and the gut microbiota remains limited. Animal studies have shown that butyrate alleviates inflammatory injury to the intestinal mucosa by reducing neutrophil recruitment, decreasing the production of inflammatory cytokines, and limiting NET formation in the intestinal mucosa. These findings highlight the important role of the gut microbiota in regulating neutrophil function.

Along the gut–lung axis, the effects of gut microbial dysbiosis on pulmonary inflammation may be mediated primarily through indirect mechanisms involving immune cells. Although direct evidence remains limited as to whether gut-derived neutrophils can migrate to the lungs through the circulation and directly trigger asthma exacerbations, their potential indirect contribution to pulmonary inflammation has attracted increasing attention. This effect may occur through the modulation of adaptive immune responses, particularly the function of T-cell subsets. Gut-primed Th17 cells can migrate to the lungs through lymphatic or circulatory routes. These cells promote a pro-inflammatory shift in the local immune microenvironment and, under certain conditions, amplify Th2-type inflammatory responses (152), thereby facilitating neutrophil recruitment and activation. In a persistent airway inflammatory microenvironment, activated neutrophils can release chromatin and granule proteins to form NETs. Although NETs have a protective role in antifungal defense, such as the clearance of *Candida albicans*, excessive NETs formation can disrupt local microbial homeostasis, sustain chronic inflammatory responses, and contribute to the development and exacerbation of asthma (153). Probiotic-based interventions provide a new perspective for modulating neutrophilic inflammation within the gut–lung axis. For example, the probiotic strain MRx0004 has been reported to alleviate severe asthma-associated airway inflammation by downregulating neutrophil-related pro-inflammatory cytokines and chemokines. This effect reduces neutrophil migration to the lungs and decreases eosinophil infiltration (154).

In summary, neutrophils are key effector cells in non-type 2 airway inflammation, and NETs formation plays an important role in amplifying airway inflammation. However, the precise mechanisms by which gut microbial dysbiosis regulates neutrophil-associated immune responses through the gut–lung axis remain to be further elucidated.

### 4.3 Mucosal barrier dysfunction

Beyond metabolic and immune regulation, mucosal barrier integrity is essential for preventing the aberrant translocation of microorganisms and their products and for maintaining local homeostasis in both the gut and the lungs. Although the gut and lung differ substantially in their anatomical structure and physiological functions, they share important similarities in embryonic origin, mucosal organization, and immune defense. Both the intestine and airways form defensive barriers composed of a single epithelial cell layer, a mucus layer, and the mucosal immune system. These barriers serve as key interfaces through which the host continuously interacts with the external environment. A growing body of evidence suggests that mucosal barrier dysfunction is an important intermediate mechanism linking gut microbiota dysbiosis to the development and progression of pediatric asthma (155, 156).

A healthy gut microbiota maintains the structural and functional integrity of the intestinal barrier by promoting epithelial cell proliferation, stimulating mucus secretion, and upregulating the expression of tight junction proteins (157). Gut microbiota dysbiosis can weaken these protective mechanisms and increase epithelial permeability. Barrier dysfunction allows the translocation of microbial metabolites, pathogen-associated molecular patterns, and inflammatory mediators, thereby inducing systemic inflammation and immune activation (158). The resulting pro-inflammatory state may promote the development of allergic diseases, including asthma. SCFAs, particularly butyrate, contribute to the maintenance of intestinal barrier homeostasis (91). Butyrate serves as an energy source for intestinal epithelial cells and upregulates the expression of mucins, antimicrobial peptides, and tight junction proteins. These effects are mediated in part through the stabilization of HIF-1α activity (159) and the activation of transcription factors such as STAT3 (160) and SP1 (161). Systemic inflammatory signals triggered by intestinal barrier disruption can affect the immune status of distal organs through the circulation, thereby creating a permissive environment for airway inflammation and increased asthma susceptibility. Airway epithelial cells are central components of the respiratory mucosal barrier. They restrict pathogen invasion and regulate local immune responses (157). In pediatric asthma, airway epithelial barrier dysfunction is characterized by reduced expression of tight junction proteins, impaired epithelial integrity, and weakened repair capacity (162, 163). Following barrier injury, airway epithelial cells release multiple inflammatory alarmins, including IL-25, IL-33, and thymic stromal lymphopoietin. These mediators directly activate ILC2s and amplify the Th2-type inflammatory cascade, thereby aggravating airway inflammation and hyperresponsiveness.

The gut and lung barriers are impaired in isolation and instead form an interconnected system through the gut–lung axis (164). Programmed death-ligand 1 (PD-L1) help maintain immune tolerance and homeostasis. PD-L1 may influence the composition of the gut and lung microbiota, increase the abundance of beneficial bacteria, such as *Lactobacillus*, improve intestinal barrier function, elevate SCFA levels, and reduce the lipopolysaccharide burden. These changes may help restore immune balance and alleviate airway inflammation in pediatric asthma (165). Viral infection provides further experimental evidence for gut–lung axis-mediated barrier injury. Influenza virus infection primarily disrupts tight junctions in the airway epithelium, leading to reduced transepithelial electrical resistance and increased epithelial permeability. In parallel, mice infected with influenza A virus show a lower relative abundance of acetate-producing bacteria, such as *Bacillus*, *Bifidobacterium*, and *Akkermansia*, accompanied by decreased acetate levels in the intestine and serum (166). A gut microbial community enriched in SCFA-producing bacteria can suppress the expression of multiple cytokines associated with airway remodeling, thereby reducing airway wall thickening. In IAV-infected mice, FMT or acetate supplementation can restore airway epithelial barrier function through the GPR43–AMPK signaling pathway and significantly reduce the levels of inflammatory cytokines (166). Various exogenous factors can modulate mucosal barrier function through the gut–lung axis. The protective effects of resveratrol against asthma are reflected not only in its regulation of the lung microbiota but also in its ability to reshape the gut microbiota. Through the gut–lung axis, resveratrol may promote the production of beneficial microbes and their metabolites, enhance pulmonary epithelial barrier function, and thereby alleviate airway inflammation (167). Prenatal antibiotic exposure may increase susceptibility to pediatric asthma by inducing early-life gut microbiota dysbiosis and intestinal barrier dysfunction in offspring. Through the gut–lung axis, these changes can drive abnormalities in the Th17/RORγt^+^ Treg immune program (168).

In summary, mucosal barrier dysfunction is a key hub linking gut–lung axis interactions to the development and progression of pediatric asthma. Impaired barrier function may facilitate the entry of microbial molecules and metabolites into the systemic circulation, thereby disrupting immune homeostasis and the pulmonary microenvironment. These alterations may establish a self-reinforcing pathological network involving the microbiota, mucosal barriers, and the immune system. A deeper understanding of the cross-organ mechanisms underlying coordinated gut and lung barrier dysfunction may help reveal the systemic immunological nature of pediatric asthma. It may also provide an important theoretical basis for early prevention and intervention strategies targeting microbiota modulation and barrier protection.

### 4.4 Multi-axis interactions

The development and progression of pediatric asthma involve coordinated dysfunction across multiple biological systems. The well-established gut–lung axis serves as a central pathway linking early-life microbial colonization to the development of pediatric asthma and plays a critical role in airway immunity. However, increasing evidence suggests that pediatric asthma is not driven by a single organ axis. Instead, it is regulated by multiple cross-organ networks, such as the gut–brain–lung axis and the gut–bone–lung axis, as illustrated in **Figure 3**.

**Figure 3:**
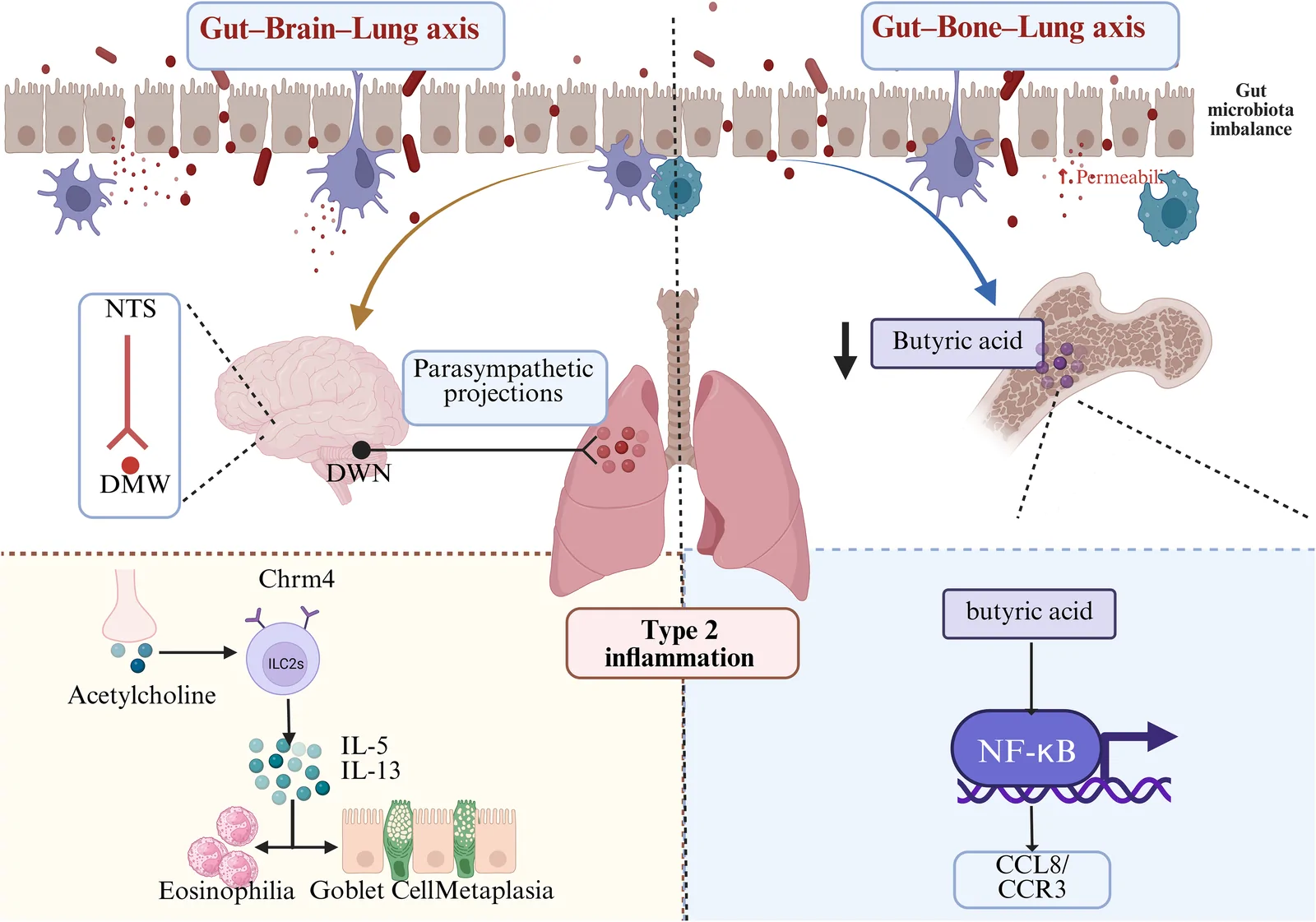
Multi-axis regulation of type 2 airway inflammation in pediatric asthma. The gut–brain–lung axis and gut–bone–lung axis jointly regulate pulmonary immune homeostasis through neural signaling and immunometabolic pathways. Dysregulation of these axes can promote the recruitment of multiple immune cell types to the lung and amplify type 2 inflammatory responses.

Within the gut–brain–lung axis, the gut microbiota can influence airway immunity through both metabolite-mediated and neural signaling pathways. In 2000, Borovikova et al. demonstrated in an endotoxin challenge model that vagus nerve-mediated parasympathetic signaling can systemically suppress the release of IL-1β, IL-6, and TNF-α from inflammatory cells through acetylcholine. This finding provided foundational evidence for the cholinergic anti-inflammatory pathway (169). Building on this concept, recent studies have further shown that food intake-induced intestinal stimulation can transmit afferent signals to the brainstem through the vagus nerve and activate parasympathetic centers. Subsequent efferent signaling to the lung promotes acetylcholine release from nerve terminals, which activates ILC2s via muscarinic acetylcholine receptor M4, leading to increased secretion of IL-5 and IL-13 and exacerbation of airway inflammation (170). This food intake-induced neuroimmune regulatory mechanism reflects organ-specific neuroimmune communication and highlights the central role of the gut–brain–lung axis in the immunoregulation of pediatric asthma.

The gut–bone–lung axis may also contribute to the pathogenesis of pediatric asthma apart from neural regulation. As a major site of immune cell generation and inflammatory memory, the bone marrow can be regulated by the gut microbiota and its metabolic signals (171). Intelectin-1 (ITLN1) has emerged as a potential regulator of asthma pathogenesis. He et al. found that ITLN1 deficiency induces gut microbiota dysbiosis and reduces butyrate production. This reduction weakens butyrate-mediated inhibition of NF-κB signaling in the bone marrow, leading to activation of the CCL8/CCR3 axis and remodeling the differentiation and migration of bone marrow-derived CD4^+^ T cells (172). These bone marrow-programmed immune cells are subsequently mobilized to the lungs, where they enhance type 2 inflammatory responses and exacerbate asthma-related phenotypes. This process highlights the key regulatory role of the gut–bone marrow–lung axis in pediatric asthma.

The gut–lung axis associated with pediatric asthma does not operate in isolation. It is embedded within a multi-axis interaction network involving the gut microbiota, nervous system, bone marrow immunity, and pulmonary inflammation. This systems-level perspective may help clarify the immunological heterogeneity of pediatric asthma and provide a theoretical basis for early intervention strategies and precision therapy.

## 5 Gut microbiota-targeted therapeutic strategies

Current clinical treatment for asthma still relies largely on long-term pharmacological therapies, such as corticosteroids and bronchodilators. However, prolonged use of these medications may have potential long-term effects on growth, development, and immune function in children. Current evidence indicates that the gut microbiota can contribute to the development and progression of asthma through the gut–lung axis. The underlying mechanisms mainly involve alterations in gut microbiota-derived metabolites, disruption of immune homeostasis, and dysfunction of the epithelial barrier (173). Based on the gut–lung axis concept, gut microbiota-targeted interventions, including probiotics and related microbial therapeutics, dietary modulation, and FMT, aim to restore gut microbial ecological balance, as shown in **Figure 4**. These strategies may provide new therapeutic approaches for asthma.

**Figure 4:**
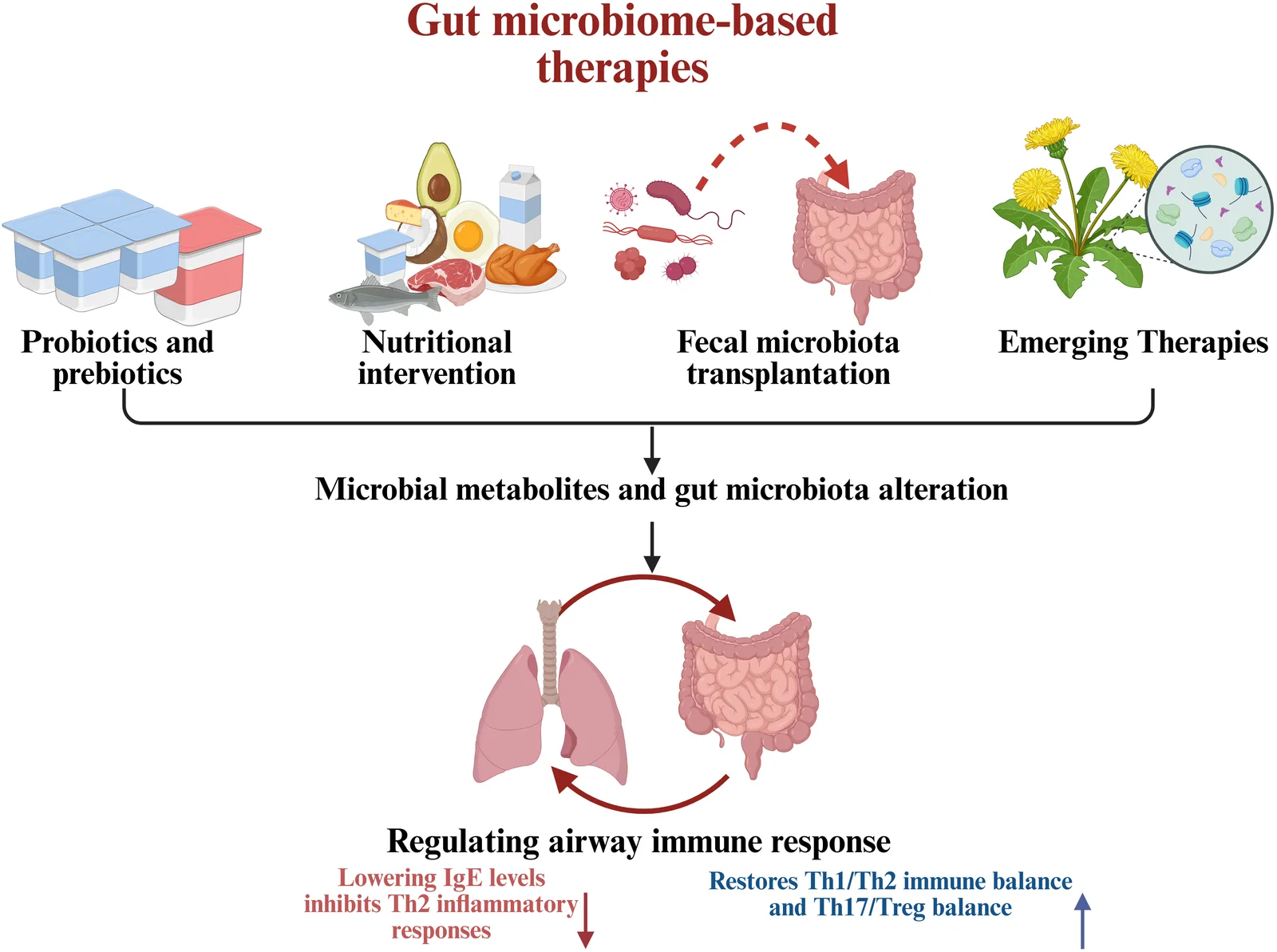
Gut microbiome-based therapeutic strategies for pediatric asthma. Probiotics and prebiotics, health-promoting nutritional interventions, FMT, and modernized traditional Chinese medicine-based therapies may represent potential therapeutic approaches for pediatric asthma.

### 5.1 Probiotic and prebiotic

Probiotics are live microorganisms that, when administered in adequate amounts, confer health benefits on the host. The most commonly used probiotics include *Lactobacillus* and *Bifidobacterium* (174). A growing body of evidence suggests that probiotics can act as upstream modulators of host–microbe interactions by correcting gut microbial dysbiosis, restoring immune homeostasis, and reducing the release of harmful metabolites. Through the gut–lung axis, probiotics may influence asthma progression and have therefore become an important focus in adjunctive therapy for pediatric asthma.

Probiotics may modulate pulmonary immune responses and airway inflammation through the gut–lung axis by reshaping the gut microbiota and its metabolic functions. In a murine model of allergic asthma, oral administration of *Bifidobacterium animalis* subsp. *lactis* CCFM1274 can increase the abundance of intestinal *Bifidobacterium* and reshape gut tryptophan metabolism (123). In addition, probiotic supplementation can promote the production of intestinal SCFAs, thereby reducing neutrophil-mediated airway inflammation in the lung (175). Beyond modulating microbial composition and metabolite production, probiotics may also directly regulate host immune responses. Preclinical studies have shown that intervention with *Lactobacillus rhamnosus* can enhance gut microbiota stability, accompanied by improved airway function and restoration of the Th1/Th2 immune balance (176). The moonlighting glyceraldehyde-3-phosphate dehydrogenase protein may exert airway-protective effects through its glycolytic activity by reversing M2-like macrophage polarization and reducing Th2-cell activation (177). In addition, oral probiotic administration can modulate the TLR4/NF-κB signaling pathway and reduce AhR expression and eosinophil infiltration. This process is accompanied by decreased levels of IgE and Th2-related cytokines (178). These preclinical studies suggest that probiotics may modulate the gut–lung axis through interconnected pathways involving the gut microbiota, microbial metabolites, immune regulation, and pulmonary inflammation. Clinical studies have reported mixed but generally favorable effects of probiotics in pediatric asthma. Studies have shown that infants at risk of asthma may exhibit transient gut microbiota dysbiosis in early life. In children at high risk of asthma, the relative abundance of bacterial genera such as *Lachnospira*, *Veillonella*, *Faecalibacterium*, and *Rothia* is significantly reduced, accompanied by disturbances in fecal metabolites, including acetate (95). These findings provide a biological rationale for early microbiota-targeted interventions. Subsequently, Huang et al. reported that *Lactobacillus* supplementation could improve clinical outcomes in asthma (179) by enhancing symptom control and reducing the frequency of acute exacerbations (180). Interestingly, maternal probiotic supplementation during pregnancy does not appear to reduce the risk of allergic diseases in children, but it is associated with recurrent wheezing at 24 months of age (181). Therefore, probiotic supplementation may also play an important role in preventing the development of asthma. A three-year longitudinal randomized controlled trial showed that formula supplemented with *Lactobacillus rhamnosus* GG reduced the incidence of asthma in children with cow’s milk allergy (182). In addition, probiotics may exert synergistic anti-inflammatory effects when used as adjunctive therapy (183). Probiotics have been widely investigated for their potential benefits in asthma management, but their effects remain uncertain. Evidence from randomized controlled trials remains insufficient to support the use of probiotics for preventing childhood asthma or other allergic diseases (184). Systematic reviews and meta-analyses of randomized controlled trials found that probiotic supplementation during the perinatal period or early life did not significantly reduce the risk of childhood asthma among high-risk infants (185–187). Heterogeneity across studies may be attributable to differences in probiotic strains, doses, and the timing of intervention, as well as variations in participant age, baseline gut microbiota composition, asthma phenotypes and endotypes, and sample size. Therefore, the clinical effects of probiotics are likely to be both strain-specific and population-dependent. Future randomized controlled trials should adopt more precisely targeted designs based on specific asthma phenotypes and microbiota profiles.

Prebiotics are non-digestible dietary components that can be selectively utilized by gut microorganisms. As substrates for beneficial bacteria, they help shape an intestinal environment that favors the growth and activity of probiotic strains, which may strengthen their physiological effects (188, 189). Preclinical studies have shown that prebiotics can reduce airway hyperresponsiveness by suppressing the secretion of Th2-associated pro-inflammatory cytokines. Blanco-Pérez et al. showed that dietary fiber pectin can alter the composition of the gut and lung microbiota and metabolite concentrations (190). Interestingly, the combined use of probiotics and prebiotics, also referred to as synbiotic intervention, can reduce eosinophil levels and appears to be more effective than probiotics alone in suppressing Th2-cell responses (191). These findings suggest that prebiotics and synbiotics may modulate the gut–lung axis through a pathway involving the gut microbiota, short-chain fatty acid production, and immune regulation. However, the available evidence is derived predominantly from basic and preclinical studies. A systematic review and meta-analysis showed that prebiotics and synbiotics attenuated airway hyperresponsiveness and type 2 inflammation in animal models. Human studies also suggested potential improvements in lung function, asthma control, and inflammatory outcomes; however, these findings require confirmation in further high-quality randomized controlled trials (192). Thus, despite the relatively consistent immunomodulatory and anti-inflammatory effects observed in animal studies, the optimal formulation, dosage, and timing of prebiotic interventions, as well as their effectiveness across different pediatric asthma phenotypes, remain unclear.

### 5.2 Nutritional interventions

Nutritional intervention is an important strategy for modulating the onset and progression of asthma. Previous studies have shown that dietary factors may alleviate asthma to some extent by regulating immune responses. Preclinical studies have shown that a high-fiber diet increases the production of SCFAs, suppresses type 2 immune responses and IgE-mediated inflammation, and is associated with CD8+ T-cell differentiation, thereby collectively attenuating the pathological processes of asthma (181, 193, 194). A growing body of clinical evidence suggests that dietary factors are associated with the development of childhood asthma. A cross-sectional study found that higher intakes of fruits, vegetables, vitamin E, and milk has been negatively associated with the risk of asthma. In particular, higher consumption of fruits and vegetables is associated with a reduced risk of childhood asthma and wheezing. These findings further support the hypothesis that changes in dietary patterns may contribute to the global increase in asthma and allergic diseases (195). Linoleic acid, a common omega-6 polyunsaturated fatty acid, has also been considered a potential factor involved in childhood asthma. Although the available evidence remains inconsistent, increasing attention has been directed toward its role in the development of childhood asthma (196). A cross-sectional study reported that adherence to a Mediterranean dietary pattern was associated with a lower prevalence of childhood asthma. Although some studies have yielded conflicting findings (193, 194, 197), these results still suggest a potential role of dietary patterns in asthma prevention. These inconsistencies may be attributable to differences in dietary composition and intake, age, genetic background, environmental exposures, early-life antibiotic use, and individual gut microbiota profiles. However, current clinical evidence is insufficient to establish whether the effects of dietary composition and dietary patterns on asthma are mediated through the gut–lung axis. Further mechanistic studies are therefore needed. In summary, nutritional intervention may influence childhood asthma, although current evidence remains controversial. These inconsistencies may be partly attributed to environmental factors and early-life antibiotic exposure. Nevertheless, nutritional intervention is still considered a promising adjunctive strategy for the prevention and management of childhood asthma.

### 5.3 Fecal microbiota transplantation

FMT involves the transfer of fecal microbiota from a healthy donor into the gastrointestinal tract of a patient, with the aim of restoring gut microbial homeostasis (198). Currently, the only formally approved indication for FMT is recurrent *Clostridioides difficile* infection. Its use in other conditions, including inflammatory bowel disease and irritable bowel syndrome, remains investigational (199). Based on the gut–lung axis concept, microbiota transplantation may have therapeutic potential for respiratory diseases such as asthma. Researchers found that transplantation of gut microbiota from children with allergic diseases enhanced type 2 immune responses and subsequently impaired lung function in mice (200). Further studies have shown that transplantation of healthy fecal microbiota promotes the production of intestinal SCFAs in rats and is accompanied by remodeling of the gut microbiota, thereby alleviating asthma associated with respiratory microbiota dysbiosis (82, 109). Unlike conventional single-strain administration, FMT can restore gut homeostasis by reshaping the diversity and composition of the intestinal microbiota (201). FMT might reduce pulmonary inflammation through metabolic and immune-mediated pathways, reflecting the interaction between the gut and lung microbial ecosystems. However, current evidence supporting FMT for pediatric asthma is derived primarily from animal studies, with little publicly available clinical evidence. FMT also carries potential risks, including the transmission of pathogenic microorganisms and parasites, while its long-term effects on the developing gut microbiota and immune system of children remain unclear. Therefore, the use of FMT in children should be restricted to clearly defined indications, with careful consideration of donor screening, informed consent, ethical review, and long-term safety. Future high-quality clinical studies conducted under rigorous safety and ethical oversight are needed to identify appropriate patient populations, optimize intervention protocols, and evaluate the long-term efficacy of FMT in pediatric asthma.

### 5.4 Emerging therapies

Asthma is one of the most common chronic diseases in childhood and remains associated with a substantial global burden of morbidity and mortality. Although advances have been made in modern medicine, current therapeutic approaches still have several limitations. Traditional Chinese medicine (TCM), characterized by its multicomponent and multitarget effects, has attracted increasing attention as a potential intervention for pediatric asthma. A limited number of clinical studies have explored the use of traditional Chinese herbal formulas in pediatric asthma. For example, a randomized, double-blind, controlled trial found that Ding Chuan Tang reduced airway hyperresponsiveness and improved symptom and medication scores in children with asthma (202). Yu Ping Feng San has also been clinically investigated as an adjunct to montelukast in children with asthma (203). However, these clinical studies primarily focused on outcomes such as symptoms, airway responsiveness, and lung function, without assessing changes in the gut microbiota. It therefore remains unclear whether the observed clinical effects involve modulation of the gut–lung axis. By contrast, current evidence that TCM influences asthma by modulating the gut microbiota is derived mainly from animal studies. Gu-Ben-Fang-Xiao Decoction alleviated gut microbiota dysbiosis in asthmatic mice, increased the abundance of short-chain fatty acid-producing bacteria and acetate levels, and promoted regulatory T-cell differentiation (204). Ma-Xing-Shi-Gan Decoction enriched potentially beneficial bacteria, including *Lactobacillus*, increased short-chain fatty acid production, and attenuated asthmatic inflammation through immunometabolic regulation involving the IL-17/Treg axis and HDAC9 (205). Collectively, these studies suggest that the anti-asthmatic effects of certain traditional Chinese herbal formulas may involve immune regulation mediated by the gut microbiota and its metabolites.

Research on plant-derived compounds for the treatment of asthma has advanced considerably. Rosmarinic acid modulated gut microbiota-derived metabolites and inflammatory signals, including 5-hydroxytryptamine (5-HT), short-chain fatty acids, and lipopolysaccharide (LPS), thereby influencing pulmonary Th2/ILC2-associated inflammation (206). Studies of tetrahydrocurcumin incorporating fecal microbiota transplantation experiments further supported the involvement of the gut microbiota in its attenuation of allergic airway inflammation (207). Resveratrol promoted the restoration of barrier integrity in both the lungs and gut by modulating the pulmonary and intestinal microbiota (167). Collectively, these animal studies suggest that plant-derived bioactive compounds may ameliorate asthma through modulation of the gut–lung axis; however, these mechanisms have not yet been validated in pediatric clinical studies. In addition, advances in nanomedicine have provided a new perspective on the investigation of TCM. Ginseng-derived cholesterol analogues, used as membrane components of lipid nanoparticles, enhanced the pulmonary delivery of MUC5AC-targeting siRNA and produced stronger anti-inflammatory effects (208). However, there is currently insufficient evidence that the anti-asthmatic effects of such nanomedicine-based formulations are directly related to modulation of the gut microbiota or the gut–lung axis. Their optimal dosage, long-term safety, and potential drug interactions in children also remain to be established.

Overall, TCM and their extracts offer potential advantages as multitarget interventions for asthma. Nevertheless, their use in pediatric asthma requires more rigorous clinical trials and safety assessments, particularly to determine whether the gut microbiota-related mechanisms observed in animal studies can be translated into clinical benefits for children.

## 6 Perspectives and conclusion

Childhood asthma is characterized by diverse clinical features and heterogeneous endotypes. Gut microbiota dysbiosis can disrupt host immune homeostasis and is closely associated with the onset and recurrence of asthma. Multiple factors, including genetic background, environmental exposures, excessive antibiotic use, and cesarean delivery, may disturb gut microbiota development, particularly in early life. This disruption can have long-lasting effects on immune system development and may increase the risk of asthma through the gut–lung axis. In this process, microbial metabolites, including SCFAs, may influence the clinical phenotypes and pathological processes of asthma by regulating airway immune responses. In addition, the gut microbiota may reduce airway inflammation by regulating immune cells, maintaining the integrity of intestinal and pulmonary barriers, and limiting the translocation of harmful substances. Therefore, elucidating the mechanisms by which the gut–lung axis contributes to childhood asthma may help identify potential therapeutic targets.

Nevertheless, several important limitations and knowledge gaps remain. First, most clinical studies have used cross-sectional designs, and heterogeneity in age, diet, antibiotic exposure, asthma phenotypes, and other factors further limits comparability across studies. Second, taxonomic changes alone cannot fully capture microbial function, whereas longitudinal studies and randomized controlled trials integrating metagenomics, metabolomics, immune profiling, and paired gut and airway samples remain scarce. Third, direct evidence of interactions between the gut and airway microbiota is still limited, and clinical evidence for probiotics, prebiotics, and fecal microbiota transplantation remains insufficient and inconsistent.

Future research should place greater emphasis on the early prediction and precision management of pediatric asthma. First, studies should evaluate the utility of the early-life gut microbiota and its metabolites as biomarkers for asthma risk. Key microbial taxa, metabolites, and their dynamic trajectories with stable predictive performance should be identified to develop models for early risk identification and stratification. Second, microbiota-targeted interventions should be tailored to specific asthma phenotypes and endotypes. Future studies should define the populations most likely to benefit from probiotics, prebiotics, and other microbiota-based interventions, as well as the optimal timing of treatment and predictors of therapeutic response. Building on these efforts, multi-omics approaches and single-cell sequencing could be used to elucidate how key microbial taxa and their metabolites regulate pulmonary immune responses and airway inflammation. Longitudinal cohorts and high-quality clinical trials are also needed to validate the predictive value of candidate biomarkers and assess the long-term safety and efficacy of precision microbiota-targeted interventions, thereby advancing the early identification, precise prevention, and individualized treatment of pediatric asthma.
